# Handling Data Heterogeneity in Electricity Load Disaggregation via Optimized Complete Ensemble Empirical Mode Decomposition and Wavelet Packet Transform

**DOI:** 10.3390/s21093133

**Published:** 2021-04-30

**Authors:** Kwok Tai Chui, Brij B. Gupta, Ryan Wen Liu, Pandian Vasant

**Affiliations:** 1 Department of Technology, School of Science and Technology, The Open University of Hong Kong, Hong Kong, China; 2 Department of Computer Engineering, National Institute of Technology Kurukshetra, Kurukshetra 136119, India; bbgupta@nitkkr.ac.in; 3 Department of Computer Science and Information Engineering, Asia University, Taichung 41354, Taiwan; 4 Macquarie University, Sydney, NSW 2109, Australia; 5 Hubei Key Laboratory of Inland Shipping Technology, School of Navigation, Wuhan University of Technology, Wuhan 430063, China; wenliu@whut.edu.cn; 6 Modeling Evolutionary Algorithms Simulation & Artificial Intelligence (MERLIN), Faculty of Electrical & Electronic Engineering, Ton Duc Thang University, Ho Chi Minh City 700000, Vietnam; pandianvasant@tdtu.edu.vn

**Keywords:** complete ensemble empirical mode decomposition, data heterogeneity, electricity load disaggregation, nonintrusive load monitoring, smart grid, smart meter, wavelet packet transform

## Abstract

Global warming is a leading world issue driving the common social objective of reducing carbon emissions. People have witnessed the melting of ice and abrupt changes in climate. Reducing electricity usage is one possible method of slowing these changes. In recent decades, there have been massive worldwide rollouts of smart meters that automatically capture the total electricity usage of houses and buildings. Electricity load disaggregation (ELD) helps to break down total electricity usage into that of individual appliances. Studies have implemented ELD models based on various artificial intelligence techniques using a single ELD dataset. In this paper, a powerline noise transformation approach based on optimized complete ensemble empirical model decomposition and wavelet packet transform (OCEEMD–WPT) is proposed to merge the ELD datasets. The practical implications are that the method increases the size of training datasets and provides mutual benefits when utilizing datasets collected from other sources (especially from different countries). To reveal the effectiveness of the proposed method, it was compared with CEEMD–WPT (fixed controlled coefficients), standalone CEEMD, standalone WPT, and other existing works. The results show that the proposed approach improves the signal-to-noise ratio (SNR) significantly.

## 1. Introduction

Reducing carbon emissions stemming from electricity consumption has been the leading global vision to tackle global warming, which can wreak havoc on human lives. Environmental experts have emphasized that global warming leads to severe ice and permafrost melting, which releases large amounts of methane, which has a greenhouse effect that is about 30 time more powerful than that for carbon dioxide [1]. This situation may lead to irreversible positive feedback for glacial melting if increased sea-level temperatures reach a certain threshold. This drives the vision for smart, green buildings to reduce the global carbon footprint [2].

In many countries, traditional electric grids have been changed to smart grids to address this challenge. Smart grids contribute to modernization by improving the transmission of electricity, the distribution system, and the electricity infrastructure. Various research topics have emerged, including battery energy storage [3], electrical–gas–hydrogen interconnected networks [4], and advanced metering infrastructure (AMI) [5]. Particularly in AMI, the deployment of smart meters, which support the continuous collection of electricity data in apartments and buildings, has played a crucial role in developing smart grids. Recent works estimated that the number of smart meters has reached 200 million in Europe, 96 million in China, 70 million in the USA, and 2.9 million in the UK, with a market penetration of over 50% [6,7]. This has built a solid foundation for further analysis of massive amounts of electricity data. In light of the introduction of the electricity load disaggregation (ELD) algorithm (also called nonintrusive load monitoring (NILM)), electricity data are disaggregated into electricity consumption of individual appliances, which brings valuable insight to the public, electric companies, and governments [8]. Users may benefit from three insights in particular: The first is determining which appliance is the most power-hungry so that follow-up actions can be taken to reduce electricity consumption in these appliances, and as a result, lower the electricity bill. Another insight is to verify whether there are appliances being turned on during the night or outside of office hours, resulting in electricity wastage. The third insight requires analysis between the past and current energy profiles of an appliance to evaluate whether the appliance has been degraded significantly. It could be more worthy to replace an appliance rather than continue to use it if it has been degraded. In terms of electricity users without smart meters, the demonstrated benefits in terms of electricity reduction among smart meter users provides a strong reason for electric companies to migrate from the traditional electric meter to the smart meter.

Various techniques, including signal processing, data mining, shallow learning, and deep learning, have been proposed for ELD in the literature. Readers who are interested in the details may refer to the latest state-of-the-art articles [9,10,11,12]. Researchers have devoted efforts to enhancing the ELD model from an algorithmic perspective, particularly toward deep learning approaches [13,14,15]. The advantages of deep learning compared to shallow learning have been demonstrated in large-scale datasets.

A critical review summarized 42 ELD datasets developed by the scientific community [16]. These datasets are heterogeneous in nature with varying factors such as location, type of space (e.g., residential, commercial, and industrial), electric appliance, powerline cable, AC power source, and smart meter.

The research focus of this paper is to merge heterogeneous datasets, which can provide two major advantages. It increases the amount of training data, especially when data collection is sometimes challenging for some appliances (suffering from small sample size). In addition, countries that have had more experience in the deployment of smart meters could support the quick rollout for those that have newly joined the smart meter initiative.

Section 1.1 presents a literature review of the techniques used to merge heterogeneous datasets. This is followed by the limitations of related works and the rationales of our work in Section 1.2. The research contributions of this paper are summarized in Section 1.3.

### 1.1. Literature Review

One review article [12] addressed the unsolved issue of data heterogeneity. It creates difficulty in fair performance evaluation and comparisons between heterogeneous datasets, yet about 40 performance metrics have been utilized in ELD research. Additionally, other heterogeneous features of public datasets include folder structure and file format [17]. Various approaches, such as those of Brick [18] and Blond [19], were employed to structure electricity data as a metadata schema in order to produce a summary of the characteristics of the ELD database. The discussions and investigations of data heterogeneity algorithms for ELD are limited. Algorithms in the literature were evaluated based on individual benchmark datasets instead of groups of benchmark datasets. Furthermore, discussions of folder structure, file format, and metadata schema [17,18,19] addressed how the attributes between datasets become consistent. This is not related to how heterogeneous datasets can be merged.

To the best of our knowledge, our research idea of merging heterogeneous ELD datasets is the first of its kind. We made the following query using the advanced search function in Web of Science: TS = ((nonintrusive load monitoring OR NILM OR load monitoring OR energy disaggregation OR electricity disaggregation OR electricity load disaggregation OR load disaggregation) AND (heterogeneity OR heterogeneous data OR heterogeneous OR heterogeneous datasets)). The same query was made using Scopus with the function TITLE-ABS-KEY. We read titles, abstracts, keywords, and introductions to confirm that there was no relevant work on the research topic.

It is worth noting that extra data generation from the source dataset [20,21] and data simulation [22] are not related to the topic of this research.

### 1.2. Limitations of Existing Works

The limitations of the existing works are as follows:

- No previous work has conducted research on merging heterogeneous ELD datasets.
- It is difficult to ensure fair performance evaluation and comparison between heterogeneous ELD datasets given that about 40 performance metrics were used.
- There is limited investigation of the powerline noise transformation between heterogeneous ELD datasets.

### 1.3. Major Research Contributions

The major research contributions of this research work are summarized as follows:

- It is the first of its kind to merge heterogeneous ELD datasets.
- It unifies the performance comparison of ELD models with merged heterogeneous datasets.
- An optimized complete ensemble empirical model decomposition and wavelet packet transform (OCEEMD–WPT) is proposed, which provides in-depth decomposition of electricity data and enhances the performance of powerline noise transformation.
- A feasibility study is carried out to confirm the enhancement of the deep learning model given the increased size of training data (after combining heterogeneous datasets).

## 2. Datasets and Methodology

In this section, 5 benchmark ELD datasets were selected to analyze the merger of heterogeneous datasets. This is followed by an illustration of the powerline noise transformation approach.

### 2.1. Benchmark Electricity Load Disaggregation Datasets

As mentioned above, one review article summarized 42 benchmark ELD datasets [16]. Five of these datasets were selected to exemplify the performance of the proposed powerline noise transformation approach. The selection criteria were based on country (the ELD datasets collected from different countries were highly heterogeneous) and sampling rate (high-frequency data, i.e., more than 10 kHz, were chosen, which led to complete information about the electricity data). In contract, low-frequency electricity data (e.g., 1 Hz) were aggregated; therefore, some essential characteristics may have been lost, thus lowering the performance of the ELD model.

The selected benchmark datasets were as follows: (i) reference energy disaggregation dataset (REDD) [23], (ii) United Kingdom domestic appliance-level electricity dataset (UK-DALE) [24], (iii) worldwide household and industry transient energy dataset (WHITED) [25], (iv) controlled on/off loads library dataset (COOLL) [26], and (v) laboratory for innovation and technology in embedded systems dataset (LIT) [27]. Table 1 summarizes the characteristics of the datasets, including country, number of classes, data duration, and sampling rate. WHITED [25] can be further categorized into 3 groups: Germany, Austria, and Indonesia. There were 7 datasets (one for each country) in total.

### 2.2. Overview of the Proposed Powerline Noise Transformation Approach

**Figure 1:**
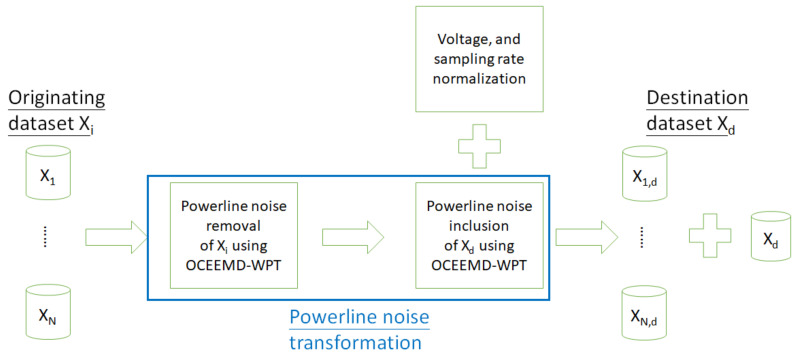
Conceptual flow of the powerline noise transformation approach via optimized complete ensemble empirical model decomposition and wavelet packet transform (OCEEMD–WPT).

The conceptual flow of the proposed powerline transformation approach is shown in Figure 1. We assume that there are M + 1 datasets, with the total number of originating datasets M = 6. The originating dataset $X_{i}=\left[X_{1},\ldots ,\;X_{M}\right]$ performs powerline noise transformation using OCEEMD–WPT (Section 2.3), including powerline noise removal from the source and powerline noise inclusion of the destination dataset $X_{d}$. The originating datasets mimicked the powerline noise of the destination dataset. The amplitude and sampling rate of $X_{i}$ are normalized to match $X_{d}$ for data homogeneity. In other words, 6 originating datasets are merged with 1 destination dataset.

### 2.3. Optimized Complete Ensemble Empirical Model Decomposition and Wavelet Packet Transform

Empirical mode decomposition (EMD) and its variants have demonstrated effectiveness in handling nonstationary and nonlinear time-series signals. They have received increasing attention based on the number of publications since 2007. Ensemble empirical mode decomposition (EEMD) was proposed in [28], which introduced Gaussian white noise (GWN) to address 2 major issues of EMD: mode mixing, which affects the further decomposition of other modes, and amplitude variation in a mode. However, EEMD has inadequacies in terms of computational cost, spectral separation of modes, and reconstruction errors. This inspired the proposal of complete ensemble empirical mode decomposition (CEEMD) [29] and improved CEEMD (ICEEMD) [30]. The controlled coefficients of the signal-to-noise ratio (SNR) in CEEMD and ICEEMD were fixed [29,30] and can be further improved by customization (via optimization).

In our work, the requirement of powerline noise transformation is to minimize the powerline noise of the originating datasets so that new powerline noise (based on destination datasets) can be added. In addition, it is desirable to maximize the noise generated by the electric appliance because it is a useful characteristic for feature extraction in the ELD model. Hence, the research problem of powerline noise transformation can be formulated as a multi-objective optimization problem called optimized complete ensemble empirical mode decomposition (OCEEMD). It helps to capture the temporal resolutions and frequency components of the signal. The signal is expressed as various intrinsic mode functions (IMFs) and a residual. Furthermore, the output of OCEEMD performs second-phase decomposition by WPT. The rationale of WPT is to emphasize time components and to characterize orthogonality, smoothness, and localization properties [31,32]. To summarize, we combined OCEEMD and WPT as OCEEMD–WPT, which captures both the time and frequency components of signals.

The mathematical formulations of OCEEMD and WPT are explained in Section 2.3.1 and Section 2.3.2, respectively.

#### 2.3.1. Optimized Complete Ensemble Empirical Model Decomposition

We consider an originating dataset $X_{i}=\left[X_{1},\ldots ,\;X_{M}\right]\forall i\in \left[1,M\right]$, where M is the total number of originating datasets. We define $X_{i}=\left[x_{i}\left(1\right),\cdots ,x_{i}\left(L_{i}\right)\right]\in R^{N}$, where $L_{i}$ is the length of $X_{i}$, which is decomposed into various IMFs and a residual using OCEEMD.

GWN $N\left(0,1\right)$ is introduced to $x_{i}\left(t\right)\forall t\in \left[1,L_{i}\right]$ with realization $j\in \left[1,J\right]$ on residual $r_{ik}\forall k\in \left[1,K\right]$, where *K* is the total number of IMFs. This GWN-masked signal is given as follows:

(1) $${\tilde{x}}_{i}^{j}\left(t\right)=x_{i}\left(t\right)+\alpha_{ik}w_{i}^{j}\left(t\right)$$

where $\alpha_{ik}$ is the controlled coefficients of the SNR to be optimized and $w_{i}^{j}\left(t\right)\;\mathrm{is}\;\mathrm{the}\;\mathrm{GWN}$. First, the first IMF ${\bar{IMF}}_{i1}\left(t\right)$ and residual $r_{i1}$ are computed:

(2) $${\bar{IMF}}_{i1}\left(t\right)=\frac{1}{J}\overset{J}{\underset{j=1}{\sum }}IMF_{i1}^{j}\left(t\right)$$

(3) $$IMF_{i1}^{j}\left(t\right)=EMD_{i1}\left({\tilde{x}}_{i}^{j}\left(t\right)\right)$$

(4) $$r_{i1}=x_{i}\left(t\right)-IMF_{i1}^{j}\left(t\right)$$

where $EMD\left(\cdot \right)$ is the basic EMD decomposition function. The decomposition is repeated with general formulas:

(5) $${\bar{IMF}}_{ik}\left(t\right)=\frac{1}{J}\overset{J}{\underset{j=1}{\sum }}EMD_{ik}\left({\tilde{r}}_{i,k-1}^{j}\left(t\right)\right)$$

(6) $${\tilde{r}}_{ik}^{j}\left(t\right)=r_{ik}\left(t\right)+\alpha_{ik}w_{i}^{j}\left(t\right)$$

(7) $$r_{ik}=r_{i,k-1}\left(t\right)-{\bar{IMF}}_{i,k-1}\left(t\right)$$

which are stopped when ${\tilde{r}}_{ik}^{j}\left(t\right)$ has one extremum. The original signal $x_{i}\left(t\right)$ can be reconstructed by all IMFs and the last residue $r_{i,final}$.

(8) $$x_{i}\left(t\right)=\overset{K}{\underset{k=1}{\sum }}{\bar{IMF}}_{ik}\left(t\right)+r_{i,final}$$

#### 2.3.2. Wavelet Packet Transform

The results of ${\bar{IMF}}_{ik}\left(t\right)$ are further decomposed and extended. The extended version of ${\bar{IMF}}_{ik}\left(t\right)$ is ${\bar{IMF}}_{ik}{\left(t\right)}_{e}\;$(of length $L_{e}$) and is given by the following:

(9) $${\bar{IMF}}_{ik}{\left(t\right)}_{e}=\left[{\bar{IMF}}_{ik,0},\cdots ,{\bar{IMF}}_{ik,L_{e}}\right]$$

(10) $$L_{e}=\left\{\begin{array}{c} length\left\{{\bar{IMF}}_{ik}\left(t\right)\right\}+2\left(L_{low}-2\right)+0\\ length\left\{{\bar{IMF}}_{ik}\left(t\right)\right\}+2\left(L_{low}-2\right)+1 \end{array}\right.\;\;\;\begin{array}{c} length\left\{{\bar{IMF}}_{ik}\left(t\right)\right\}=even\\ length\left\{{\bar{IMF}}_{ik}\left(t\right)\right\}=odd \end{array}$$

with low-pass filter $h_{low}=\left[h_{low,0},\cdots ,h_{low,L_{low}-1}\right]$ of length $L_{low}$.

The general form of the approximated WPT coefficients with $h_{low}$ is given by the following:

(11) $$a_{ik,m}=\overset{L_{low}}{\underset{n=0}{\sum }}{\bar{IMF}}_{ik,2m+n}\times h_{low,n}\forall m\in \left[0,\left(L_{e}-L_{low}\right)/2\right]$$

Likewise, the high-pass filter $h_{high}=\left[h_{high,0},\cdots ,h_{high,L_{high}-1}\right]$ of length $L_{high}$ is defined. The general form of approximated WPT coefficients with $h_{high}$ is given by the following:

(12) $$b_{ik,m}=\overset{L_{high}}{\underset{n=0}{\sum }}{\bar{IMF}}_{ik,2m+n}\times h_{high,n}\forall m\in \left[0,\left(L_{e}-L_{high}\right)/2\right]$$

For the selection of wavelets, typical Daubechies wavelets (D2–20) were selected for analysis.

As mentioned above, the controlled coefficients of the SNR $\alpha_{ik}$ must be optimized. We formulated the optimization problem as a multi-objective optimization problem with two objective functions, in which $F_{1}$ is the kurtosis and $F_{2}$ is the residual difference:

(13) $$\begin{array}{cc} Max & F_{1}=\frac{E\left\{{\left(\gamma -\bar{\gamma }\right)}^{4}\right\}}{\sigma_{\gamma }{}^{4}} \end{array}$$

(14) $$\begin{array}{cc} Min & F_{2}= \end{array}\sqrt{\frac{\sum_{m}{\left(\hat{x}\left[m\right]-x\left[m\right]\right)}^{2}}{\sum_{m}x{\left[m\right]}^{2}}}$$

where $E\left\{\cdot \right\}$ is the expected value, and $\bar{\gamma }$ and $\sigma_{\gamma }$ are the average and standard deviation, respectively, of wavelet coefficients γ.

**Figure 2:**
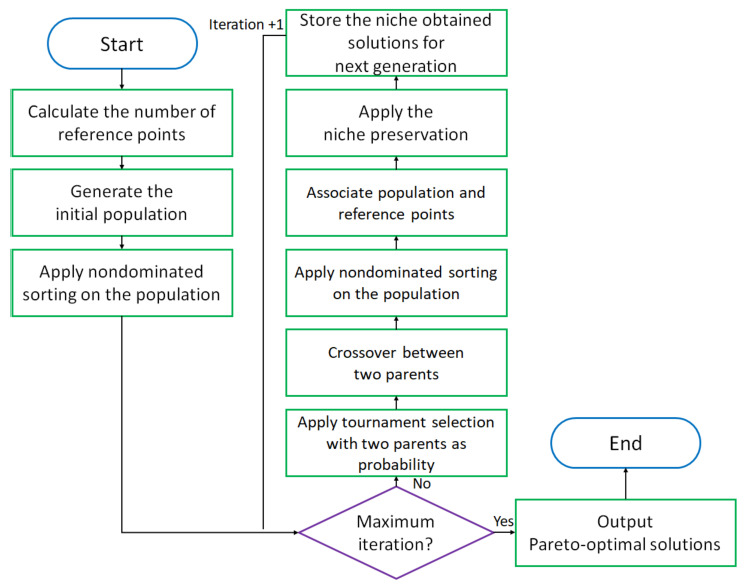
Conceptual flow of the NSGA-III-based OCEEMD–WPT.

A reference-point-based multi-objective evolutionary algorithm following the NSGA-II framework (NSGA-III) [33,34] was adopted to solve the multi-objective optimization problem. NSGA-III has advantages in solving the optimization problem with smaller population sizes, thus lowering the computation time, enhancing the diversity of the new population based on the reference points, and using adaptive allocation of reference points depending on the Pareto-optimal front. The flow of the NSGA-III-based OCEEMD–WPT is shown in Figure 2.

The pseudo-code of the NSGA-III is summarized in Algorithm 1. The reference points are predefined with locations and uniformly distributed on a hyperplane to ensure the convergence of solutions. It adopts a set of reference directions (rays starting from the original and pointing towards the reference point) to maintain the diversity among solutions. The goal of a multi-objective evolutionary algorithm is to seek a Pareto solution set that is evenly distributed, well extended, and converged. Regarding the association of the populations with reference points, there are two possibilities: (i) if only one member of the population is associated with the reference point, the reference point is ignored in the current generation and, (ii) if more than one member of the population is associated with the reference point, the member with the shortest perpendicular distance is included.
**Algorithm 1** $Training\left(X_{i}\right)$Input: Training datasets $X_{i}$Output: NSGA-III-based OCEEMD–WPT Model1. Calculate the number of reference points;2. Generate NSGA-III parameters such as population size and values of the objective functions;3. Apply non-dominated sorting on the population; **while** iterations i≤ maximum number of_iterations **do**4. Apply tournament selection with two parents in terms of probability;5. Apply crossover between two parents;6. Apply non-dominated sorting on the population;7. Associatae the populations with reference points;8. Apply the niche preservation to select individuals associated with each reference point;9. Store the niche obtained solutions for the next generation;10. *i = i + 1*;End whileModel←Pareto optimal solutions

## 3. Analysis and Comparison

To evaluate the performance of the proposed NSGA-III optimized OCEEMD–WPT approach for merging heterogeneous ELD datasets, four studies were conducted (i) on the performance of NSGA-III-optimized OCEEMD–WPT, (ii) on the contribution of NSGA-III to solving the controlled coefficients, (iii) on the contribution of merging CEEMD and WPT, and (iv) on the performance of the proposed approach in comparison to existing works merging time-series heterogeneous data.

The performance indicator of the powerline noise transformation is based on the average improvement of signal-to-noise ratio (SNR) in dB.

### 3.1. Performance Evaluation of Proposed Work

Recall that there are seven heterogeneous ELD datasets considered, as shown in Table 1. The evaluation of the proposed NSGA-III optimized OCEEMD–WPT can be formulated as seven destinations (each ELD dataset corresponds to one destination). The experiment is based on a workstation (i7-10850H 2.7–5.1 GHz CPU, NVIDIA Quadro RTX 3000 6 GB GDDR6 GPU, and 64 GB memory). The average computational times for WPT (one execution), CEEMD (one execution), and NSGA-III are 0.001 s, 0.0568 s, and 8.5 min to 1.4 h, respectively. The average improvement in SNR is summarized in Table 2.

Based on the results, there are two key observations:

- The larger the number of classes in the originated ELD dataset, the larger the average improvement in SNR.
- The larger the number of classes in the destination ELD dataset, the larger the average improvement in SNR.

### 3.2. Study on the Contribution of NSGA-III to Solving Controlled Coefficients

The optimal design of the controlled coefficients of the SNR was solved by NSGA-III and compared with the performance based on fixed controlled coefficients (without optimization) [29,30]. Table 3 summarizes the average improvement in SNR of CEEMD–WPT. It can be seen from the results that CEEMD–WPT yields a smaller average improvement in SNR compared with the proposed NSGA-III optimized OCEEMD–WPT. When attributed to the fixed controlled coefficients using CEEMD–WPT, less powerline noise can be eliminated.

Comparing the columns between Table 2 and Table 3, the proposed approach improves the average SNR by 37.3–47.4%, 43.2–53.6%, 34.0–52.9%, 30.7–50%, 34.0–54.5%, 43.1–52.5%, and 43.9–50.8% for REDD, UK-DALE, WHITED (Germany), WHITED (Austria), WHITED (Indonesia), COOLL, and LIT, respectively. This reflects the need for an optimal design of the controlled coefficients.

### 3.3. Study on the Contribution of Merging Complete Ensemble Empirical Model Decomposition and Wavelet Packet Transform

To examine the advantages of merging CEEMD and WPT as two stages of decomposition of electricity data, the performance of the average improvement in SNR using either standalone CEEMD or WPT is summarized in Table 4 and Table 5, respectively.

**Figure 3:**
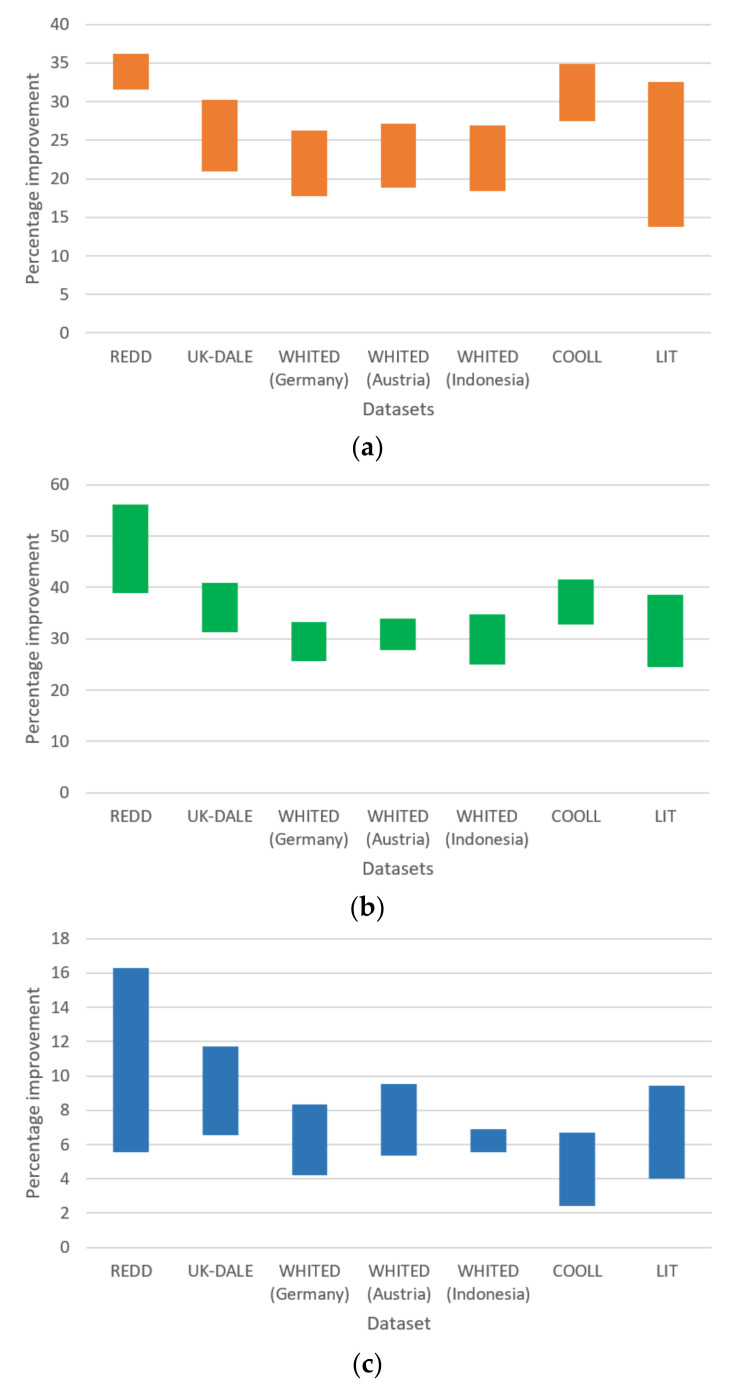
Summary on the range of percentage improvements between models: (**a**) between the proposed model and standalone CEEMD, (**b**) between the proposed work and standalone WPT, and (**c**) between standalone CEEMD and standalone WPT.

Figure 3 presents the range of percentage improvements by the proposed work compared with standalone CEEMD (Figure 3a) and standalone WPT (Figure 3b) as well as those between standalone CEEMD and standalone WPT (Figure 3c). The proposed work achieved the greatest improvements in SNR, followed by standalone CEEMD and standalone WPT.

### 3.4. Performance Comparison between the Proposed Approach and Existing Works

To the best of our knowledge, this is the first study to consider merging heterogeneous ELD datasets. Therefore, we compared the proposed approach with those in existing works on other research topics based on time-series data. Table 6 summarizes the performance of the proposed approach and those in existing works using discrete Fourier series [35], CEEMD with permutation entropy [36], and discrete Fourier transform and discrete cosine transform [37].

Compared to [35], the proposed approach enhances the SNR by 83.9–121%, 76.9–96.3%, 62.5–89.1%, 57.1–78.0%, 61.5–92.5%, 82.4–102%, and 62.5–87.2% for REDD, UK-DALE, WHITED (Germany), WHITED (Austria), WHITED (Indonesia), COOLL, and LIT, respectively. The results reveal that the proposed approach outperforms those in existing works [35,36,37]. Compared to [36], the proposed approach enhances the SNR by 56.1–71.4%, 56.8–86.0%, 47.7–73.3%, 46.7–72.1%, 46.5–85.5%, 60.3–80.9%, and 53.2–70.4%, respectively. Finally, compared to [34], the proposed approach enhances the SNR by 25.3–33.3%, 31.8–43.2%, 22.6–38.7%, 20–34.6%, 23.5–43.7%, 24–38.5%, and 26.7–35.3%, respectively.

## 4. Conclusions and Future Work

Merging ELD datasets (heterogeneous in nature) provides a larger pool of data for training ELD models. More data availability is advantageous for deep learning-based methods. In this paper, we propose an NSGA-III-based OCEEMD–WPT approach for powerline noise transformation so that heterogeneous ELD datasets can be merged, with the unification of powerline noise. Various studies determining the necessity for NSGA-III, for combining CEEMD and WPT, and for making comparisons with existing works were conducted to confirm the effectiveness of the proposed approach, which enhances the SNR significantly. The results of this research could be beneficial in shifting from total electricity consumption to consumption of individual appliances, which could possibly reduce the number of power-hungry appliances. Current work could be realized by enabling optimal tracking [38] and price control [39] strategies for heterogeneous loads. Secured control can be guaranteed using blockchain-based authentication and authorization [40], and convolutional neural network [41]. Consequently, climate change as a critical governing factor in the global hydrological cycle could be relieved [42].

Since the current work is the first to consider merging ELD datasets, there are research limitations; thus, we suggest conducting further investigations in the following areas: (i) consideration of more ELD datasets based on a summarized list of datasets from a review article [16]. (ii) evaluation of the performance of the proposed approach and existing works in low-frequency (i.e., aggregated electricity data) ELD datasets. (iii) evaluation of the performance enhancement of deep learning-based models for ELD, and (iv) exploration of alternative approaches to addressing the challenges that arise when the number of classes in the ELD datasets is small.

## Figures and Tables

**Table 1:** Summary of selected benchmark datasets: REDD, reference energy disaggregation dataset; UK-DALE, United Kingdom domestic appliance-level electricity dataset; WHITED, worldwide household and industry transient energy dataset; COOLL, controlled on/off loads library dataset; LIT, laboratory for innovation and technology in embedded systems dataset.

Dataset	Country	Number of Classes	Data Duration	Sampling Rate (kHz)
REDD [23]	USA	20	Several months	16.5
UK-DALE [24]	UK	40	Up to 2 years	16
WHITED [25]	Germany, Austria, and Indonesia	47	5 s	44.1
COOLL [26]	France	12	6 s	100
LIT [27]	Brazil	14	30 s to several hours	15

**Table 2:** Average improvement in signal-to-noise ratio (SNR) using the proposed NSGA-III-optimized OCEEMD–WPT.

	Average Improvement in SNR (dB)						
Destination	REDD	UK-DALE	WHITED (Germany)	WHITED (Austria)	WHITED (Indonesia)	COOLL	LIT
REDD [23]	N/A	10.6	11.2	11.9	10.8	7.8	8.2
UK-DALE [24]	9.3	N/A	12.5	12.7	12.3	8.5	8.8
WHITED [25] (Germany)	9.8	11.1	N/A	13.2	12.5	9.2	9.4
WHITED [25] (Austria)	9.9	11.6	13.0	N/A	12.6	9.3	9.5
WHITED [25] (Indonesia)	10.3	10.9	12.7	13.0	N/A	9.0	9.2
COOLL [26]	8.4	8.9	10.4	10.5	10.2	N/A	7.8
LIT [27]	8.7	9.2	10.6	10.8	10.5	8.1	N/A

**Table 3:** Average improvement in SNR with CEEMD–WPT (with fixed controlled coefficients).

	Average Improvement in SNR (dB)						
Destination	REDD	UK-DALE	WHITED (Germany)	WHITED (Austria)	WHITED (Indonesia)	COOLL	LIT
REDD [23]	N/A	6.9	7.8	8.3	7.6	5.3	5.7
UK-DALE [24]	6.4	N/A	9.0	9.4	8.8	5.8	6.1
WHITED [25] (Germany)	6.7	7.6	N/A	10.1	9.0	6.2	6.4
WHITED [25] (Austria)	7.1	8.1	9.7	N/A	9.4	6.5	6.6
WHITED [25] (Indonesia)	7.5	7.4	9.3	9.8	N/A	5.9	6.1
COOLL [26]	5.7	6.1	6.8	7.0	6.6	N/A	5.4
LIT [27]	6.0	6.3	7.2	7.5	6.9	5.6	N/A

**Table 4:** Average improvement in SNR with standalone CEEMD.

	Average Improvement in SNR (dB)						
Destination	REDD	UK-DALE	WHITED (Germany)	WHITED (Austria)	WHITED (Indonesia)	COOLL	LIT
REDD [23]	N/A	5.3	6.5	6.9	6.2	4.0	4.4
UK-DALE [24]	4.7	N/A	7.4	7.7	7.2	4.3	4.6
WHITED [25] (Germany)	5.0	6.2	N/A	8.5	7.6	4.8	5.2
WHITED [25] (Austria)	5.4	6.7	8.1	N/A	7.9	5.1	5.8
WHITED [25] (Indonesia)	5.7	6.1	7.9	8.2	N/A	4.6	4.9
COOLL [26]	4.2	4.9	5.4	5.6	5.2	N/A	4.3
LIT [27]	4.5	5.1	5.7	5.9	5.6	4.2	N/A

**Table 5:** Average improvement in SNR with standalone WPT.

	Average Improvement in SNR (dB)						
Destination	REDD	UK-DALE	WHITED (Germany)	WHITED (Austria)	WHITED (Indonesia)	COOLL	LIT
REDD [23]	N/A	4.9	6.0	6.3	5.8	3.8	4.2
UK-DALE [24]	4.1	N/A	7.1	7.2	6.8	4.1	4.4
WHITED [25] (Germany)	4.3	5.7	N/A	7.9	7.2	4.5	5.0
WHITED [25] (Austria)	4.9	6.0	7.6	N/A	7.4	4.9	5.3
WHITED [25] (Indonesia)	5.4	5.5	7.4	7.6	N/A	4.4	4.7
COOLL [26]	3.9	4.6	5.1	5.3	4.9	N/A	4.1
LIT [27]	4.2	4.8	5.4	5.6	5.3	4.1	N/A

**Table 6:** Average improvement in SNR using the proposed approach and those in existing works [35,36,37].

	Originating Dataset													
	REDD		UK-DALE		WHITED (Germany)		WHITED (Austria)		WHITED (Indonesia)		COOLL		LIT	
Destination Dataset	Improvement in SNR presenting in a format of							Proposed				[35]		
								[36]				[37]		
REDD [23]	N/A	N/A	10.6	5.4	11.2	6.3	11.9	6.8	10.8	6.1	7.8	4.0	8.2	4.5
	N/A	N/A	5.7	7.4	7.0	8.4	7.4	9.0	6.7	8.3	4.5	6.0	4.9	6.3
UK-DALE [24]	9.3	4.2	N/A	N/A	12.5	7.5	12.7	8.0	12.3	7.3	8.5	4.2	8.8	4.7
	5.5	7.0	N/A	N/A	8.1	9.9	8.3	10.2	7.6	9.4	4.7	6.6	5.3	6.9
WHITED [25] (Germany)	9.8	4.5	11.1	6.1	N/A	N/A	13.2	8.4	12.5	7.6	9.2	4.7	9.4	5.2
	5.9	7.5	6.7	8.2	N/A	N/A	9.0	11.0	8.2	9.6	5.4	6.8	5.7	7.2
WHITED [25] (Austria)	9.9	5.2	11.6	6.5	13.0	8.0	N/A	N/A	12.6	7.8	9.3	5.1	9.5	5.7
	6.3	7.9	7.4	8.8	8.8	10.6	N/A	N/A	8.6	10.2	5.8	7.5	6.2	7.5
WHITED [25] (Indonesia)	10.3	5.6	10.9	5.9	12.7	7.6	13.0	7.9	N/A	N/A	9.0	4.6	9.2	5.0
	6.6	8.2	6.5	7.9	8.5	10.2	8.8	10.6	N/A	N/A	5.3	6.5	5.4	6.8
COOLL [26]	8.4	4.0	8.9	4.9	10.4	5.5	10.5	5.9	10.2	5.3	N/A	N/A	7.8	4.8
	4.9	6.3	5.5	6.5	6.0	7.5	6.1	7.8	5.5	7.1	N/A	N/A	4.7	6.1
LIT [27]	8.7	4.5	9.2	5.2	10.6	5.9	10.8	6.2	10.5	5.5	8.1	4.4	N/A	N/A
	5.5	6.7	5.8	6.9	6.2	8.0	6.4	8.2	6.0	7.5	5.0	6.3	N/A	N/A

## Data Availability

No new data were created or analyzed in this study. Data sharing is not applicable to this article.

## References

[1] Masyagina O.V., Menyailo O.V. (2020). The impact of permafrost on carbon dioxide and methane fluxes in Siberia: A meta-analysis. Environ. Res..

[2] Stergiou C.L., Psannis K.E., Gupta B.B. (2020). IoT-based Big Data secure management in the Fog over a 6G Wireless Network. IEEE Internet Things J..

[3] Alsaidan I., Khodaei A., Gao W. (2017). A comprehensive battery energy storage optimal sizing model for microgrid applications. IEEE Trans. Power Syst..

[4] Tostado-Véliz M., Arévalo P., Jurado F. (2021). A comprehensive electrical-gas-hydrogen Microgrid model for energy management applications. Energy Convers. Manag..

[5] Chi H.R., Tsang K.F., Chui K.T., Chung H.S.H., Ling B.W.K., Lai L.L. (2016). Interference-mitigated ZigBee-based advanced metering infrastructure. IEEE Trans. Ind. Informat..

[6] Chen Y., Martínez-Ortega J.F., Castillejo P., López L. (2019). A homomorphic-based multiple data aggregation scheme for smart grid. IEEE Sens. J..

[7] Wang Y., Chen Q., Hong T., Kang C. (2019). Review of smart meter data analytics: Applications, methodologies, and challenges. IEEE Trans. Smart Grid.

[8] Chui K.T., Lytras M.D., Visvizi A. (2018). Energy sustainability in smart cities: Artificial intelligence, smart monitoring, and optimization of energy consumption. Energies.

[9] Gopinath R., Kumar M., Joshua C.P.C., Srinivas K. (2020). Energy management using non-intrusive load monitoring techniques-State-of-the-art and future research directions. Sustain. Cities Soc..

[10] Yuan X., Han P., Duan Y., Alden R.E., Rallabandi V., Ionel D.M. (2020). Residential Electrical Load Monitoring and Modeling–State of the Art and Future Trends for Smart Homes and Grids. Electr. Power Compon. Syst..

[11] Nalmpantis C., Vrakas D. (2019). Machine learning approaches for non-intrusive load monitoring: From qualitative to quantitative comparation. Artif. Intell. Rev..

[12] Pereira L., Nunes N. (2018). Performance evaluation in non-intrusive load monitoring: Datasets, metrics, and tools—A review. Wiley Interdiscip. Rev. Data Min. Knowl. Discov..

[13] Kong W., Dong Z.Y., Wang B., Zhao J., Huang J. (2019). A practical solution for non-intrusive type II load monitoring based on deep learning and post-processing. IEEE Trans. Smart Grid.

[14] Houidi S., Fourer D., Auger F. (2020). On the use of concentrated time–frequency representations as input to a deep convolutional neural network: Application to non intrusive load monitoring. Entropy.

[15] Faustine A., Pereira L., Klemenjak C. (2020). Adaptive weighted recurrence graphs for appliance recognition in non-intrusive load monitoring. IEEE Trans. Smart Grid.

[16] Iqbal H.K., Malik F.H., Muhammad A., Qureshi M.A., Abbasi M.N., Chishti A.R. (2021). A critical review of state-of-the-art non-intrusive load monitoring datasets. Electr. Power Syst. Res..

[17] Pereira M., Velosa N., Pereira L. (2019). dsCleaner: A Python Library to Clean, Preprocess and Convert Non-Intrusive Load Monitoring Datasets. Data.

[18] Balaji B., Bhattacharya A., Fierro G., Gao J., Gluck J., Hong D., Johansen A., Koh J., Ploennigs J., Agarwal Y. (2018). Brick: Metadata schema for portable smart building applications. Appl. Energy.

[19] Kriechbaumer T., Jacobsen H.A. (2018). BLOND, a building-level office environment dataset of typical electrical appliances. Sci. Data.

[20] Mukaroh A., Le T.T.H., Kim H. (2020). Background Load Denoising across Complex Load Based on Generative Adversarial Network to Enhance Load Identification. Sensors.

[21] Chen K., Zhang Y., Wang Q., Hu J., Fan H., He J. (2020). Scale-and context-aware convolutional non-intrusive load monitoring. IEEE Trans. Power Syst..

[22] Klemenjak C., Kovatsch C., Herold M., Elmenreich W. (2020). A synthetic energy dataset for non-intrusive load monitoring in households. Sci. Data.

[23] Kolter J.Z., Johnson M.J. REDD: A public data set for energy disaggregation research. Proceedings of the Workshop on Data Mining Applications in Sustainability.

[24] Kelly J., Knottenbelt W. (2015). The UK-DALE dataset, domestic appliance-level electricity demand and whole-house demand from five UK homes. Sci. Data.

[25] Kahl M., Haq A.U., Kriechbaumer T., Jacobsen H.A. Whited-a worldwide household and industry transient energy data set. Proceedings of the 3rd International Workshop on Non-Intrusive Load Monitoring.

[26] Picon T., Meziane M.N., Ravier P., Lamarque G., Novello C., Bunetel J.C.L., Raingeaud Y. (2016). COOLL: Controlled on/off loads library, a public dataset of high-sampled electrical signals for appliance identification. arXiv.

[27] Renaux D., Linhares R., Pottker F., Lazzaretti A., Lima C., Neto A.C., Campaner M. Designing a novel dataset for non-intrusive load monitoring. Proceedings of the 2018 VIII Brazilian Symposium on Computing Systems Engineering.

[28] Wu Z., Huang N.E. (2009). Ensemble empirical mode decomposition: A noise-assisted data analysis method. Adv. Adapt. Data Anal..

[29] Torres M.E., Colominas M.A., Schlotthauer G., Flandrin P. A complete ensemble empirical mode decomposition with adaptive noise. Proceedings of the 2011 IEEE International Conference on Acoustics, Speech and Signal Processing.

[30] Colominas M.A., Schlotthauer G., Torres M.E. (2014). Improved complete ensemble EMD: A suitable tool for biomedical signal processing. Biomed. Signal Process. Control.

[31] Plaza E.G., López P.N. (2018). Application of the wavelet packet transform to vibration signals for surface roughness monitoring in CNC turning operations. Mech. Syst. Signal Process..

[32] Islam M.M., Kim J.M. (2019). Automated bearing fault diagnosis scheme using 2D representation of wavelet packet transform and deep convolutional neural network. Comput. Ind..

[33] Deb K., Jain H. (2014). An evolutionary many-objective optimization algorithm using reference-point-based nondominated sorting approach, part I: Solving problems with box constraints. IEEE Trans. Evol. Comput..

[34] Jain H., Deb K. (2014). An evolutionary many-objective optimization algorithm using reference-point based nondominated sorting approach, part II: Handling constraints and extending to an adaptive approach. IEEE Trans. Evol. Comput..

[35] Bahaz M., Benzid R. (2018). Efficient algorithm for baseline wander and powerline noise removal from ECG signals based on discrete Fourier series. Australas. Phys. Eng. Sci. Med..

[36] Liu F., Cai M., Wang L., Lu Y. (2019). An ensemble model based on adaptive noise reducer and over-fitting prevention LSTM for multivariate time series forecasting. IEEE Access.

[37] Singhal A., Singh P., Fatimah B., Pachori R.B. (2020). An efficient removal of power-line interference and baseline wander from ECG signals by employing Fourier decomposition technique. Biomed. Signal Process. Control.

[38] Anand S.C., Baldi S. Optimal tracking strategies for uncertain ensembles of thermostatically controlled loads. Proceedings of the 2020 IEEE 16th International Conference on Control & Automation.

[39] Zou S., Chen Z., Lygeros J. Price Control for Heterogeneous Thermostatically Controlled Loads in Communication and Computation Delay Environments. Proceedings of the 2019 IEEE 58th Conference on Decision and Control.

[40] Esposito C., Ficco M., Gupta B.B. (2021). Blockchain-based authentication and authorization for smart city applications. Inf. Process Manag..

[41] Li D., Deng L., Gupta B.B., Wang H., Choi C. (2019). A novel CNN based security guaranteed image watermarking generation scenario for smart city applications. Inf. Sci..

[42] Kumar N., Poonia V., Gupta B.B., Goyal M.K. (2021). A novel framework for risk assessment and resilience of critical infrastructure towards climate change. Technol. Forecast. Soc. Chang..

